# Abdominal cocoon syndrome. A rare cause of mechanical intestinal obstruction: A case report

**DOI:** 10.1016/j.ijscr.2023.107875

**Published:** 2023-01-06

**Authors:** Mehwish Mehmood, Ahsan Ali Mirza, Gummadi Sai Sree, Naga Praneeth Vakkalagadda, Hassan Mumtaz

**Affiliations:** a Fauji Foundation Hospital, Pakistan; b PAEC Hospital, Pakistan; c Government General hospital, Guntur medical college, Guntur, India; d Clinical Research Associate: Maroof International Hospital, Public Health Scholar: Health Services Academy, Pakistan

**Keywords:** Abdominal cocoon syndrome, Intestinal obstruction, Diagnosis, laparotomy

## Abstract

**Introduction and importance:**

Abdominal Cocoon Syndrome (ACS) also known as Idiopathic.

Sclerosing Peritonitis, is a rare cause of Mechanical Intestinal Obstruction.

**Case presentation:**

We present a 59-year-old man with severe intestinal blockage symptoms for three days. Rectum had a noticeable amount of abdominal fat. To rule out the more common causes of mechanical blockage, a CT scan revealed the presence of a rare condition called Cocoon Syndrome, which necessitated exploratory laparotomy and adhesiolysis surgery. After the surgery, the patient was declared stable and was released from the hospital.

**Clinical discussion:**

The diagnosis of a tuberculous abdominal cocoon before surgery is a real challenge.

**Conclusion:**

Recognizing and understanding this entity, as well as the usual radiological findings, may help in its appropriate treatment.

## Highlights

• This case report found a thick fibro collagenous membrane completely or partially encases the small intestinal loops, leading to Abdominal Cocoon Syndrome.
• Recognizing and understanding this entity, as well as the usual radiological findings, may help in its appropriate treatment
• In most cases, surgery is the preferred treatment method. An early biopsy of the peritoneum can often rule out the need for surgery in most cases.

## 1 Introduction

Abdominal cocoon syndrome, also known as sclerosing peritonitis, is a rare disease that causes obstructions in the small intestine. Chronic fibrosis encapsulates (ACS) were first described in 1907 by Owtschinnikow and renamed in 1978 by Foo et al. [1].

It is distinguished by “total or partial encasement of the small gut in a fibro-collagenous cocoonlike sac” in addition to “extensive intrinsic small bowel adhesions.” Abdominal cocoon syndrome occurs when *Mycobacterium Bovis* is present in raw cow's milk [2].

Variations in primary and acquired (secondary) forms are the most common. Female adolescence is particularly prevalent in tropical and subtropical countries. Peritoneal inflammation and irritation are the most common side effects, leading to peritoneal fibrogenesis [3].

The most common symptom of an abdominal cocoon is a blockage of the intestines, whether or not a large mass accompanies them. A definitive diagnosis can only be made following a laparotomy and adhesion lysis to remove the obstruction. A preoperative diagnosis may still be made by doctors familiar with the disease and its radiologic symptoms [4]. Our case report is in accordance with SCARE guidelines 2020 [5].

## 2 Case presentation

A 59-year-old man with a three-year history of hypertension presented with a one-day history of growing TP increasing stomach pain and frequent projectile vomiting, followed by absolute constipation for one day and abdominal distension six hours before presentation. No history of fever, malaise, jaundice, hematemesis, or melena was available. There was no family history of TB. He had a similar attack previously six months ago, which was conservatively controlled and he was discharged. He was alert, oriented, afebrile, and mildly dehydrated upon inspection. Abdominal examination revealed tenderness throughout the abdomen and increased bowel sounds. However, there was a palpable abdominal swelling. Rectum was found to be empty on digital rectal examination (DRE). He was originally managed by maintaining nothing by mouth, using O2 inhalation, nasogastric aspiration, and Foley catheterization. Intravenous fluid resuscitation was initiated, as well as empiric antibiotics. Blood tests revealed normal hepatic and renal profiles during laboratory workup. His coagulation profile was slightly abnormal, and he had metabolic alkalosis as determined by ABGs. LDH and CEA levels were within normal limits. Simple abdominal X-rays indicated Distended small bowel loops with varying air-fluid levels and an absence of rectum gas shadow, as shown in Fig. I. Ultrasound of the abdomen was inconclusive.

**Fig. I:**
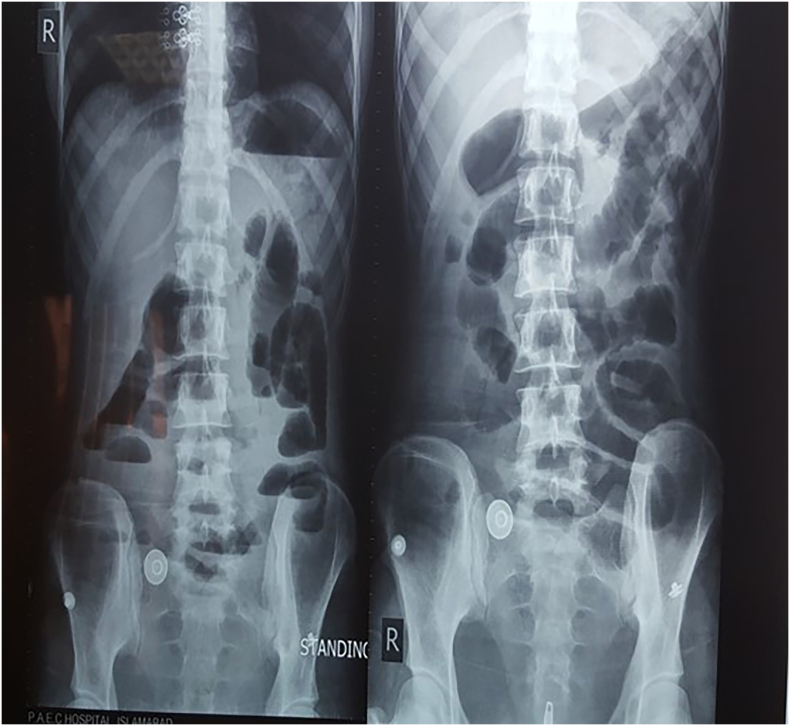
Dilated small bowel loops multiple air fluid levels with the absence of gas in the rectum.

Contrast-Enhanced Abdominal CT scan (CECT) showed Dilated fluid-filled Small Bowel loops (mainly Jejunal) with max. diameter of 4.5 cm, arranged strangely. Loops were enclosed in a thick membrane while a transition zone appears outside the enclosing membrane. A provisional diagnosis of Abdominal Cocoon Syndrome as given in Fig. II. Keeping in mind the CT scans, consultant opted for exploratory laparotomy.

**Fig. II:**
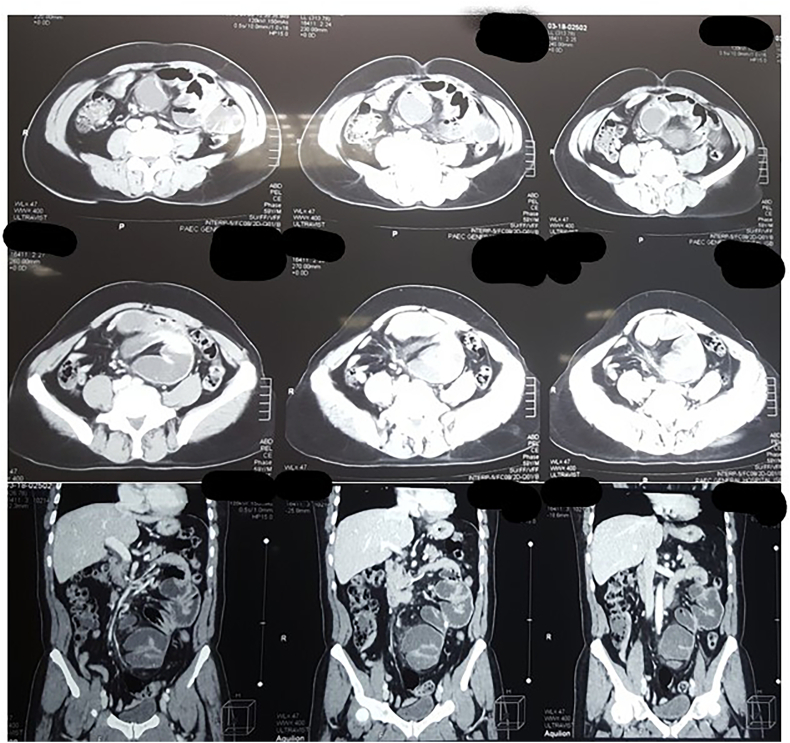
Ct scan showing abdominal cocoon syndrome.

Preparation for exploratory laparotomy was performed on the patient by the Consultant Surgeon.

A midline incision was used to expose the abdomen and visualize the interior anatomy. Numerous jejuna and proximal ileum loops were discovered to be in a strong, thick, white membrane (Fig. IIIa), which consisted of two layers. Within the sac's thick, fibrous outer membrane was a thin, avascular membrane. The liver, stomach, and jejunal/ileal loops were all covered in a dense sac. The Encasing Membrane was opened, adhesiolysis was performed, and the membrane was completely removed as Illustrated in Fig. IIIa, b & c. The entire colon was healthy except for two locations where the gut wall had thinned out and friable, necessitating primary bowel repair. Additionally, the membrane was sent for histopathology. The abdomen was cleansed completely with normal saline and then closed with a drain friable.

**Fig. III:**
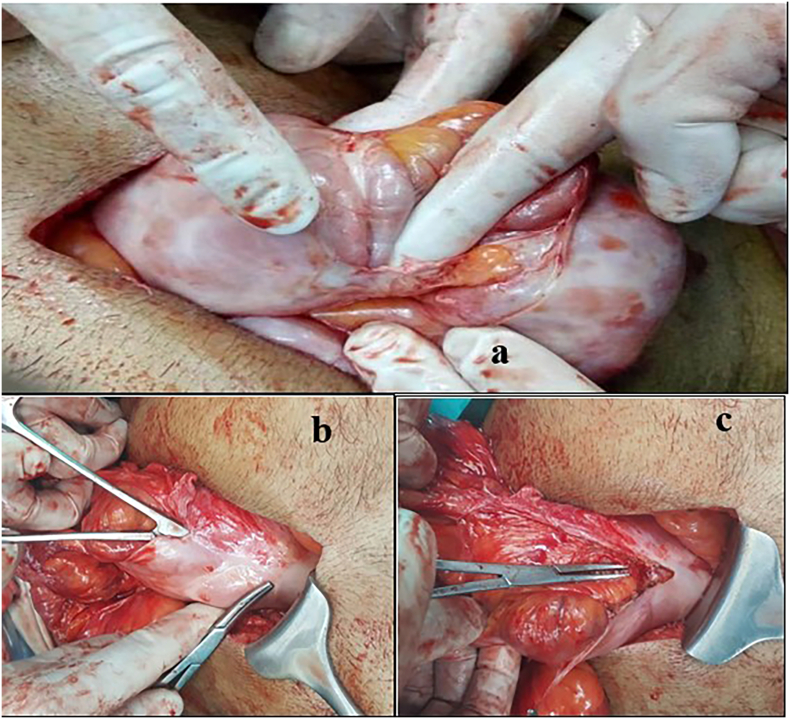
a: Intraoperative findings showing abdominal cocoon. b: Tough whitish membrane encasing the whole bowel. c: Adhesion lysis of membrane encasing jejunal loops.

The patient was released without difficulties on the fourth postoperative day and advised bed rest for a week. His osteopath's report revealed the following: Peritoneal tissue: A kind of fibrosis that is densely hyalinized, with mild chronic inflammation and dystrophic calcification. One of the surgery main complications is “bowel injury” and extra care should be taken in consideration.

## 3 Discussion

Small bowel loop encasement in a thick fibrous collagenous membranous sac is an uncommon clinical disease known as Abdominal Cocoon Syndrome.

The small bowel is the primary organ affected by ACS, which is an uncommon condition. Fibrous adhesions around the intestine create a cocoon or sac that causes acute or persistent intestinal obstruction [6]. In 1978, Foo et al. published the first definition of ACS [1].

The etiology has been attributed to a wide range of factors. Some of the most frequent treatments are peritoneal dialysis, abdominopelvic TB, post-renal transplantation povidone‑iodine, and long-term beta-blocker usage, as well as cancers including GIST and neuroendocrine tumors. There have also been reports of connections between the abdominal cocoon and cryptorchidism, omental hypoplasia's, gut malrotation, and Treitz's hernia [7]. However, idiopathic causes account for the vast majority of instances.

Many hypotheses indicating pathogenesis of EPS/Cocoon syndrome were proposed but an epithelial-to-mesenchymal cell transition occurs in the peritoneal lining, and this results in the development of a thick membrane. Also, the most accepted view is that any agent that causes irritation of the mesothelial layer and induces serositis, or single severe or multiple episodes of peritonitis resulting in mesothelial loss, predisposes the peritoneum to fibrogenesis [8]. Mesothelial denudation, angiogenesis, interstitial fibrosis, and vascular sclerosis are among the morphologic alterations. Reduced peritoneal lining absorptive surface area is one of the functional alterations [9].

In most cases, Clinical Features remains symptomless until there is an obstruction to the gut, whereas others exhibit clinical characteristics such as abdominal discomfort and distention, nausea, and signs and symptoms of the acute abdomen or small intestinal obstruction. The patient has vomit, weight loss, and has a non-tender abdominal tumor that is palpable in some instances.

To correctly diagnose EPS, several radiological tests may be used, beginning with plain X-rays of the abdomen, which may show multiple air-fluid levels and clumping of the small intestinal loops, as well as peritoneal or intestinal wall calcifications [10]. Diagnosis by ultrasound abdomen depends mainly on the expertise of radiologists. Cauliflower-like distended bowel loops connected by an indistinct mesentery are a common feature in ACS. An echogenic membrane surrounding the intestinal loops causes the “sandwich sign”, which may also be observed [11]. However, Contrast-enhanced computerized tomography (CECT) is the diagnostic investigation for EPS. It continues to be the gold standard for diagnosis [12].

Findings on CECT maybe thickening of the abdomen and intestines including small intestine, Ascites with lymphadenopathy. In the visceral and muscular layers, calcification of the wall or peritoneum, particularly around the capillaries.

Clumped bowel loops connected by a thin mesentery cause the cauliflower sign [13]. For this reason, MDCT is preferred because it provides a more complete picture by depicting disease progression and subtle radiologic changes [14].

The treatment of EPS is a complicated problem that requires knowledge of the etiopathogenesis and the patient's stage at the time of presentation.

In the current medical treatment of EPS, immunosuppressive and antifibrotic drugs are employed to treat the inflammatory and fibrotic changes at the peritoneal membrane level. Abdominal cocoons are still treated with laparotomy, membrane excision, and an adhesiolytic. Several surgical procedures are available for individuals who show indications of intestinal blockage or who have been identified intraoperatively with ACS. The membrane is partially removed, adhesions are removed, and the anastomosis and resection anastomosis with a covering ileostomy are performed [15].

An exploratory laparotomy or a combination of techniques may be used depending on the patient's individual situation. As well as eliminating the underlying cause, Idiopathic Cocoon Syndrome patients should have surgery to remove the intestinal membrane and remove thick adhesions between the intestinal loops. The recurrence risk is low when the intestinal surface membrane can be fully eliminated. It may be as simple as injecting an anti-adhesive substance between the intestinal loops before sealing the abdomen to prevent postoperative adhesions [16].

Cocoon abdomen, according to a case study from Aghakhan University, is one of the rarest causes of chronic intestinal blockage, and its presentation with sepsis is much more unusual. It is an unexpected finding at the moment of laparotomy in cases of emergency presentation. If the bowel appears healthy and alive upon examination, the recommended therapy is removal of the sclerosing membrane and repositioning of the gut [17].

## 4 Conclusion

Rarely, a thick fibro collagenous membrane completely or partially encases the small intestinal loops, leading to Abdominal Cocoon Syndrome. Here we describe a rare instance that was seen at our facility and treated appropriately. Recognizing and understanding this entity, as well as the usual radiological findings, may help in its appropriate treatment.

## Consent for publication

Written informed consent was obtained from the patient for publication of this case report and accompanying images. A copy of the written consent is available for review by the Editor-in Chief of this journal.

## Ethical approval

This case report was approved by the ethics committee of PAEC Hospital, ref. no PAEC/ERC/037.

## Funding

The authors declare that they had no financial support for the development of this research.

## Author contribution

1. Concept: Mehwish Mehmood, **Ahsan ali Mirza**.
2. **Writing – review and editing**: Hassan Mumtaz.
3. **Writing –original draft**: Gummadi Sai Sree, Naga Praneeth Vakkalagadda.

## Guarantor

Hassan Mumtaz.

## Research registration number

NA.

## Provenance and peer review

Not commissioned, externally peer-reviewed.

## Declaration of competing interest

The authors declare no any conflict of interest in this paper.
